# Supplementary material for: Resolving the full spectrum of human genome variation using Linked-Reads
Source: Genome Res. 2019 Apr;29(4):635–45. doi: 10.1101/gr.234443.118 (PMC6442396; doi:10.1101/gr.234443.118)
Supplement: Supplemental Material [file supp_gr.234443.118_Supplemental_longranger-2.2.zip › longranger-2.2.tar.bz2!/lr2.2-public/lib/stan/cmdstan/stan/doc/api/html/classstan_1_1agrad_1_1var-members.html]

Stan: Member List


|  |  |
| --- | --- |
|  | Stan  2.5.0  probability, sampling & optimization |


- Main Page
- Related Pages
- Namespaces
- Classes
- Files

- Class List
- Class Index
- Class Hierarchy
- Class Members

All Classes Namespaces Files Functions Variables Typedefs Enumerations Enumerator Friends Macros Pages

- stan
- agrad
- var

stan::agrad::var Member List

This is the complete list of members for stan::agrad::var, including all inherited members.

|  |  |  |
| --- | --- | --- |
| adj() const | stan::agrad::var | inline |
| grad(std::vector< var > &x, std::vector< double > &g) | stan::agrad::var | inline |
| is\_uninitialized() | stan::agrad::var | inline |
| operator\*() | stan::agrad::var | inline |
| operator\*=(const var &b) | stan::agrad::var | inline |
| operator\*=(const double b) | stan::agrad::var | inline |
| operator+=(const var &b) | stan::agrad::var | inline |
| operator+=(const double b) | stan::agrad::var | inline |
| operator-=(const var &b) | stan::agrad::var | inline |
| operator-=(const double b) | stan::agrad::var | inline |
| operator->() | stan::agrad::var | inline |
| operator/=(const var &b) | stan::agrad::var | inline |
| operator/=(const double b) | stan::agrad::var | inline |
| operator<<(std::ostream &os, const var &v) | stan::agrad::var | friend |
| Scalar typedef | stan::agrad::var |  |
| val() const | stan::agrad::var | inline |
| var(vari \*vi) | stan::agrad::var | inlineexplicit |
| var() | stan::agrad::var | inline |
| var(bool b) | stan::agrad::var | inline |
| var(char c) | stan::agrad::var | inline |
| var(short n) | stan::agrad::var | inline |
| var(unsigned short n) | stan::agrad::var | inline |
| var(int n) | stan::agrad::var | inline |
| var(unsigned int n) | stan::agrad::var | inline |
| var(long int n) | stan::agrad::var | inline |
| var(unsigned long int n) | stan::agrad::var | inline |
| var(unsigned long long n) | stan::agrad::var | inline |
| var(long long n) | stan::agrad::var | inline |
| var(float x) | stan::agrad::var | inline |
| var(double x) | stan::agrad::var | inline |
| var(long double x) | stan::agrad::var | inline |
| vi\_ | stan::agrad::var |  |


---

[ Stan Home Page ]

*© 2011–2014,
Stan Development Team.*
